# Cytokine storm promoting T cell exhaustion in severe COVID-19 revealed by single cell sequencing data analysis

**DOI:** 10.1093/pcmedi/pbac014

**Published:** 2022-05-23

**Authors:** Minglei Yang, Chenghao Lin, Yanni Wang, Kang Chen, Yutong Han, Haiyue Zhang, Weizhong Li

**Affiliations:** Zhongshan School of Medicine, Sun Yat-sen University, Guangzhou 510080, China; Zhongshan School of Medicine, Sun Yat-sen University, Guangzhou 510080, China; Zhongshan School of Medicine, Sun Yat-sen University, Guangzhou 510080, China; Zhongshan School of Medicine, Sun Yat-sen University, Guangzhou 510080, China; Zhongshan School of Medicine, Sun Yat-sen University, Guangzhou 510080, China; Zhongshan School of Medicine, Sun Yat-sen University, Guangzhou 510080, China; Zhongshan School of Medicine, Sun Yat-sen University, Guangzhou 510080, China; Center for Precision Medicine, Sun Yat-sen University, Guangzhou 510080, China; Key Laboratory of Tropical Disease Control of Ministry of Education, Sun Yat-Sen University, Guangzhou 510080, China

**Keywords:** COVID-19, immune exhaustion, cytokine storm, single-cell sequencing data analysis, T cell, immune checkpoint

## Abstract

**Background:**

Evidence has suggested that cytokine storms may be associated with T cell exhaustion (TEX) in COVID-19. However, the interaction mechanism between cytokine storms and TEX remains unclear.

**Methods:**

With the aim of dissecting the molecular relationship of cytokine storms and TEX through single-cell RNA sequencing data analysis, we identified 14 cell types from bronchoalveolar lavage fluid of COVID-19 patients and healthy people. We observed a novel subset of severely exhausted CD8 T cells (Exh T_CD8) that co-expressed multiple inhibitory receptors, and two macrophage subclasses that were the main source of cytokine storms in bronchoalveolar.

**Results:**

Correlation analysis between cytokine storm level and TEX level suggested that cytokine storms likely promoted TEX in severe COVID-19. Cell–cell communication analysis indicated that cytokines (e.g. CXCL10, CXCL11, CXCL2, CCL2, and CCL3) released by macrophages acted as ligands and significantly interacted with inhibitory receptors (e.g. CXCR3, DPP4, CCR1, CCR2, and CCR5) expressed by Exh T_CD8. These interactions formed the cytokine–receptor axes, which were also verified to be significantly correlated with cytokine storms and TEX in lung squamous cell carcinoma.

**Conclusions:**

Cytokine storms may promote TEX through cytokine-receptor axes and be associated with poor prognosis in COVID-19. Blocking cytokine-receptor axes may reverse TEX. Our finding provides novel insights into TEX in COVID-19 and new clues for cytokine-targeted immunotherapy development.

## Introduction

The coronavirus disease 2019 (COVID-19) pandemic has caused >500 million infections and 6.2 million deaths by 27th April 2022 (WHO data at https://www.who.int/emergencies/diseases/novel-coronavirus-2019). The over-production of pro-inflammatory cytokines known as a cytokine storm resulted in immune system disorders including constitutional symptoms, systemic inflammation, and multiorgan dysfunction that increase the risk of death.^1,2^ It is recognized that cytokine storms may be associated with CD8 T cell exhaustion (TEX) in severe COVID-19.^3–5^ Maraviroc, a C-C chemokine receptor 5 (CCR5) antagonist, which may reverse lymphoid exhaustion and alter the cell trafficking of inflammatory cells, has been tested in clinical trials for severe COVID-19 (ClinicalTrials.gov Identifier: NCT04435522, NCT04441385, and NCT04475991). CCR5 inhibition could also reduce cytokine storms, increase CD8 T cells, and decrease SARS-CoV-2 RNA abundance in severe COVID-19.^6^ However, the interplay mechanism between cytokine storms and TEX remains unclear. For example, how do cytokine storms affect TEX and further impact clinical outcomes? Answers to these questions are urgently needed for the development of new drugs and therapies that act by decreasing cytokine storms and reactivating exhausted T cells.

TEX also occurs in many chronic viral infections, including human immunodeficiency virus (HIV),^7,8^ hepatitis C virus (HCV),^9^ and hepatitis B virus (HBV).^10^ Similarly, severe acute respiratory syndrome coronavirus 2 (SARS-CoV-2) can potentially destroy the function of CD4 T cells and promote the excessive activation and possible subsequent exhaustion of CD8 T cells.^11^ Patients with severe COVID-19 might have cytokine storm syndrome or hyperinflammatory syndrome characterized by fulminant and fatal hypercytokinaemia with multiorgan failure.^1^ Recent reports also indicated that the increasing pro-inflammatory cytokine or chemokine responses damaged the homeostasis of the immune system, resulting in cytokine storm syndromes in patients with severe SARS-CoV-2 infection.^1^ Systemically elevated cytokines are cardiotoxic and possibly lead to myocardial injury. A minority of patients have the cardiovascular manifestations of COVID-19 disease, such as viral myocarditis, associated with a high risk of mortality.^12^

In this study, we aimed to dissect the molecular relationship between cytokine storms and TEX, and to determine the prognostic significance of cytokine storms. We first profiled and characterized immune cell types from bronchoalveolar lavage fluid (BALF) of COVID-19 patients and healthy people through single-cell RNA sequencing data analysis. We then focused the analysis on a subset of severely exhausted CD8 T cells that co-expressed several inhibitory receptors and significantly interacted with macrophages through the cytokines released by macrophages. Further, we found that cytokine–receptor interaction axes (e.g. CCL2-CCR2, CCL3-CCR1, CCL3-CCR5, CCL4-CCR5, CCL4L2-VSIR, and CCL3L1-CCR1) observed in severe COVID-19 were significantly correlated with TEX. Our study tried to provide new insights into the interplay mechanism between cytokine storms and TEX, and to suggest potential cytokine-targeted drug development for COVID-19.

## Materials and methods

### Source data retrieval

The raw count matrices of single-cell sequencing data for BALF (GSE145926)^13^ and peripheral blood mononuclear cells (PBMC) (GSE150728)^14^ were downloaded from NCBI Gene Expression Omnibus (GEO) (https://www.ncbi.nlm.nih.gov/geo/). A total of 12 BALF samples were collected from 6 severe COVID-19 patients, 3 moderate COVID-19 patient samples, and 3 healthy controls. The 13 PBMC samples included 3 severe COVID-19 patient samples, 4 moderate COVID-19 patient samples, and 6 healthy controls. The clinical information of the patient samples and the definition of disease severity are summarized in Table 1.^13,14^ For the PBMC and BALF samples, most patients belonging to the severe group were males or >60 years old. Detailed information of the source data is listed in Supplementary Table 1, see online supplementary material. The tools used in this study are summarized in Supplementary Table 2, see online supplemetary material.

**Table 1.** Clinical information of the patient samples used in this study.

Donor	Gender	Age (years)	Severity	Disease	Tissue	GEO Sample ID (GSM)
M1	Male	36	Moderate	COVID-19	BALF	GSM4339769
M2	Female	37	Moderate	COVID-19	BALF	GSM4339770
M3	Male	35	Moderate	COVID-19	BALF	GSM4339772
S1	Male	62	Severe	COVID-19	BALF	GSM4339773
S2	Male	66	Severe	COVID-19	BALF	GSM4339771
S3	Male	38	Severe	COVID-19	BALF	GSM4339774
S4	Female	65	Severe	COVID-19	BALF	GSM4475051
S5	Female	36	Severe	COVID-19	BALF	GSM4475052
S6	Male	46	Severe	COVID-19	BALF	GSM4475053
HC1	Female	38	Healthy	COVID-19	BALF	GSM4475048
HC2	Male	24	Healthy	COVID-19	BALF	GSM4475049
HC3	Male	22	Healthy	COVID-19	BALF	GSM4475050
C1A	Male	60–69	Moderate	COVID-19	PBMC	GSM4557327
C1B	Male	60–69	Severe	COVID-19	PBMC	GSM4557328
C2	Male	40–49	Moderate	COVID-19	PBMC	GSM4557329
C3	Male	60–69	Severe	COVID-19	PBMC	GSM4557330
C4	Male	30–39	Severe	COVID-19	PBMC	GSM4557331
C5	Male	50–59	Moderate	COVID-19	PBMC	GSM4557332
C7	Male	20–29	Moderate	COVID-19	PBMC	GSM4557333
H1	Female	40–49	Healthy	COVID-19	PBMC	GSM4557334
H2	Male	40–49	Healthy	COVID-19	PBMC	GSM4557335
H3	Female	30–39	Healthy	COVID-19	PBMC	GSM4557336
H4	Male	40–49	Healthy	COVID-19	PBMC	GSM4557337
H5	Male	40–49	Healthy	COVID-19	PBMC	GSM4557338
H6	Male	30–39	Healthy	COVID-19	PBMC	GSM4557339

### Quality control on single-cell RNA-seq data

We performed quality control for the raw count data of each single-cell RNA-seq sample using Seurat (v4). To ensure the consistency of the analysis, we applied the same criteria to all the samples of healthy, moderate, and severe groups. During acute infection, T cell metabolic activity is increased, possibly resulting in mitochondrial gene expression.^15^ Therefore, the mitochondrial gene percentage, a key parameter for filtering cells, was set at 20%. In addition, the cells that expressed <200 genes or >6000 genes were also discarded. For gene filtering, genes that were expressed in <10 cells were removed from the final count matrix. After the quality control, 84 114 cells from BALF and 48 507 PBMC were retained for further analysis.

### Clustering, annotation, and identification of cell types

**Figure 1.**
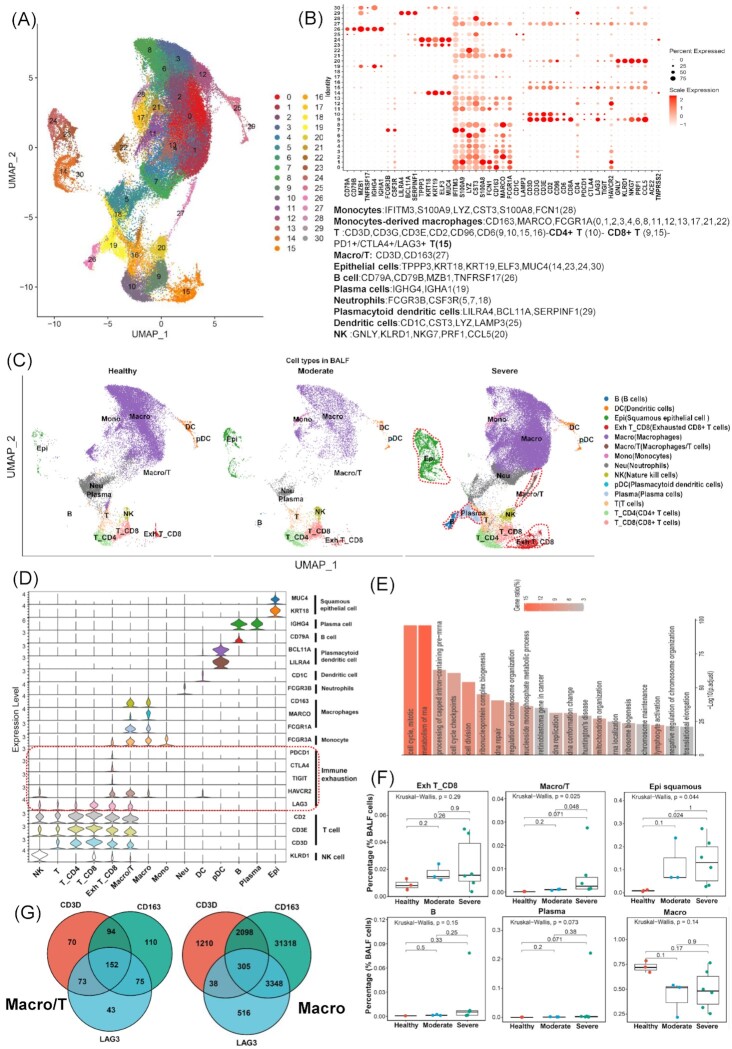
Identification of cell types in BALF and cell–cell interaction of exhausted CD8 T cells. (**A**) Overview of the cell clusters in BALFs (*n* = 12) derived from across healthy people (*n* = 3), moderate (*n* = 3), and severe (*n* = 6) COVID-19 patients. (**B**) Dot plots show the expression level of canonical markers representing cell types and genes related to SARS-CoV-2 entry into cells. Cell types and their corresponding genes are listed at the bottom. Each dot is colored according to the scale expression and sized by the percentage of cells in a cluster (row) which expresses a given gene (column). (**C**) UMAP plots of BALF colored by cell types across three disease states including healthy (left panel), moderate (middle panel), and severe (right panel). (**D**) Violin plots show the expression of canonical maker genes (rows) which represent the distinct cell types (columns) and multiple inhibitory receptors indicating TEX. (**E**) Top 20 pathway/process enrichment analysis of all highly expressed genes in exhausted CD8 T cells; adjusted *P* value < 0.0001 (hypergeometric test, adjusted *P*-values obtained by the Benjamini–Hochberg procedure). (**F**) The cell percentages of Exh T_CD8, Macro/T, Epi squamous, B, Plasma, and Macro cells among healthy controls (*n* = 3), moderate patients (*n* = 3), and severe patients (*n* = 6). The Kruskal–Wallis H test was used for comparing the three disease states. (**G**) Venn diagrams display the numbers of cells concurrently expressing CD3D, CD163, and LAG3 in Macro/T (left panel) and Macro (right), respectively. B, B cells. DC, dendritic cells. Epi, squamous epithelial cells. Exh T_CD8, exhausted CD8 T cells. Macro, macrophages. Macro/T, a mixture of macrophages and T cells. Mono, monocytes. Neu, neutrophils. NK, nature kill cells. pDC, plasmacytoid dendritic cells. Plasma, plasma cells. T, T cells. T_CD4, CD4 T cells. T_CD8, CD8 T cells.

Due to the heterogeneity of BALF and PBMCs, we adopted two different strategies of clustering and analysis. For BALF, each dataset was normalized and identified with the top 2000 variable features by the “vst” method in Seurat (v4). Then, to correct batch effects among the samples, all datasets were integrated into a filtered gene-barcode matrix using the “FindIntegrationAnchors” and “IntegrateData” functions in Seurat. Dimensionality reduction was performed by principal component analysis (PCA). Then we used the JackStraw procedure^16^ wrapped in Seurat and the Elbow plot to evaluate how many principal components (PCs) should be chosen to perform further clustering analysis. According to the evaluation result of the PCs, we tried a series of PC numbers (40–60) (Supplementary Fig. 2A, see online supplementary material) to cluster cells and subsequently identified the best PC number based on the expression of the well-known cell type (T cell) markers by using the “VlnPlot” function. To our prior knowledge, T cells have similar expression patterns and should be clustered together during cell clustering. When the PCs number was set as 50, the T cell cluster was clearly observed in our cell clusters (Fig. 1C). Therefore, the top 50 PCs were used to conduct uniform manifold approximation and projection (UMAP) for the visualization of single-cell groups in 2D space. Meanwhile, the top 50 PCs were also used to construct a shared nearest-neighbour graph (SNN; FindNeighbors), followed by graphical clustering (FindClusters) with the graph-based modularity-optimization algorithm Louvain for community detection. The resolution was a flexible parameter varying with the total number of cells during cell clustering. Larger resolution makes more clusters. To cluster 84 114 cells of BALF, we tested different resolution values from 0.8 to 1.5 and then set 1.2 as the best resolution. Furthermore, specific markers in each cluster were identified by the “FindAllMarkers” function and each cluster was assigned to a known cell type using the combination of SingleR automatic annotation and manual annotation according to canonical genes from CellMarker.^17^ Then we again applied the “FindAllMarkers” function to obtain highly expressed genes of each cell type. Finally, to understand the molecular biological characteristics of exhausted CD8 T cells, we applied Metascape (https://metascape.org/) to perform a pathway enrichment analysis on all highly expressed genes with the average log2 (fold-change) of >0.25 and adjusted *P* value < 0.05.^18^

For the PBMC data analysis, all filtered datasets were first merged into a gene-barcode matrix with the “merge” function in Seurat. The gene-barcode matrix was first normalized by using the ‘LogNormalize’ method in Seurat (v4) with default parameters. The top 2000 variable genes were then identified using the ‘vst’ method in the Seurat “FindVariableFeatures” function. Using the same method in the BALF data analysis, we tested a series of PC numbers (15–20) (Supplementary Fig. 3A, see online supplementary material) and finally determined 18 as the best PC number for cell clustering. So, dimension reduction was performed based on the top 2000 variable genes with PCA and then UMAP was performed on the top 18 PCs. We also tested the various values of resolution ranging from 0.5 to 1.0, and found 0.7 made the finest clustering for the PBMC data. Finally, we assigned each cluster to a known cell type in the same manner for BALF.

Additionally, we also reviewed and tried other deep-learning-based methods^19–24^ to cluster the single cell RNAseq data to validate our results of cell clustering. To identify the cell types enriched in BALF and PBMC of the severe COVID-19 patient group, we applied the Kruskal–Wallis test to compare the percentages of each cell type among severe, moderate, and healthy groups.

### Cell–cell interaction analysis

To establish a cell–cell interaction network via ligand–receptor interactions, we applied CellPhoneDB^25^ to the single cell raw count matrices of BALF and PBMC respectively in severe COVID-19 patients. The ligand–receptor pairs that were expressed in <10% of cells were discarded. To identify the significant cell–cell interactions, we performed permutation tests between two cell types mediated by a specific ligand–receptor pair. The permutation tests were based on the mean gene expression of the ligand from one cell type and the corresponding receptor from another cell type. According to the protocol of CellPhoneDB, the smaller the *P*-value, the more reliable the resulting ligand-receptor complexes. Therefore, to reduce the false positives, *P*-value  < 0.01 was considered statistically significant. Finally, we constructed a cell–cell crosstalk network based on the number of cell–cell interactions.

### Association analysis of cell types related to strong cytokine storm and immune exhaustion scores for BALF

To explore cytokine storm-related cell types, we collected the cytokine storm genes (*CXCL10, CCL3, CCL2, IL2, IL7, CSF3, TNF*, and *IL6*) from a previous study^1^ and defined cytokine storm scores using the “AddModuleScore” function in Seurat (v4). We then used the sigmoid function to normalize cytokine storm scores to a range from 0 to 1 and plotted the normalized scores in a UMAP plot. We defined the immune exhaustion score based on six inhibitory receptors (*PDCD1, CTLA4, LAG3, BTLA, TIGIT*, and *HAVCR2*) through the same definition method as for cytokine storm score. We performed the Mann–Whitney rank test for each cell type's score versus all other types’ score, and the Kruskal–Wallis test for every cell type score comparison among severe, moderate, and healthy groups. Pearson correlation was applied to correlate the median cytokine storm scores of macrophages with the median immune exhaustion scores of (exhausted) CD8 T cells across three groups for BALF.

### Re-clustering the macrophages of BALF to identify subclasses related to cytokine storm

To investigate which macrophage subclasses were the main source of cytokines, we separated macrophages from all the cell types of BALF by the “SplitObject” function. Then we tested a series of resolution values (0.1–0.9) to re-cluster macrophages and combined the expression of cytokine storm genes in the identified clusters to determine the best resolution value. Then we re-clustered these cells into 7 subclasses by using the “FindClusters” function with a resolution of 0.2. We tried UMAP andt-distributed stochastic neighbor embedding (tSNE) to visualize the 7 subclasses of macrophages and found tSNE made more clear clustering 2D space. Following the same approach for determining the previously mentioned PC parameter, we set 45 as the best PC number to cluster. We performed tSNE on the top 45 PCs to visualize the distribution of macrophage subclasses among severe, moderate, and healthy groups. Then we calculated the expression level of cytokine storm-related genes across 7 macrophage subclasses and identified differently expressed genes in each subclass using the “FindAllMarkers” function. We compared the percentages of 7 subclasses among three disease states using the Kruskal–Wallis test. We conducted the pathway and process enrichment analyses based on the top 100 highly expressed genes in subclasses enriched in the severe group.

### Statistical analysis

Statistical discrete analyses were performed in R (version 3.5.1, http://www.r-project.org). The Kaplan–Meier estimate and log-rank testing were used to perform survival analysis. We used the Wilcoxon rank-sum test to analyse the correlation between continuous variables and categorical variables. We used the ‘corr.test’ function wrapped in psych (https://CRAN.R-project.org/package = psych) for Pearson analysis and *P*-value adjustment.

## Results

### Identifying severe CD8 TEX and relevant cell types in BALF of COVID-19 patients via single-cell transcriptomic analysis

To explore TEX in the bronchoalveolar immune microenvironment, we conducted an analysis on the scRNA-seq data derived from BALF cells of patients with moderate and severe COVID-19, and of healthy controls. The original sequencing data and the clinical features are available from the publicly reported study by Liao *et al*.^13^ Graph-based clustering identified 30 clusters (Fig. 1A and B). Based on the expression with at least two canonical genes from multiple COVID-19 single-cell studies,^13,17,26^ we assigned each cluster to 14 cell types (Fig. 1B and C). In particular, we defined the exhausted CD8 T cell (Exh T_CD8) cluster based on the elevated expression of multiple inhibitory receptors (*TIGIT, LAG3, PDCD1, CTLA4*, and *HAVCR2*) (Fig. 1D and Supplementary Table 3, see online supplementary material), and this cluster has not been reported by previous studies.^13^ Gene annotation and enrichment analysis of all differently expressed genes in the exhausted CD8 T cell cluster versus other cell clusters (Supplementary Table 3) suggested that these T cells might have enhanced the metabolism of RNA and cell division (Fig. 1E). Notable differences of cell type distribution could be observed based on the UMAP among the different infection states (healthy, moderate, and severe) (Fig. 1C). Exh T_CD8, squamous epithelial cells (Epi), and the mixture of macrophage and T cells (Macro/T) were visually enriched in the severe COVID-19 group (Fig. 1C). In addition, we used several deep-learning-based methods^19–24^ to cluster the cells and found scvi-tools produced a comparable result. Specifically, 45 clear clusters were identified (Supplementary Fig. 1A). When applying the cell annotation from the Seurat result to these clusters, most of the cells for each cell type were clustered together in UMAP (Supplementary Fig. 1B).

We compared the percentage of each cell type among three disease states, and found that Exh_T CD8, Macro/T, and Epi cells were enriched in the severe group and the other cell types were not enriched in any group (Fig. 1F and Supplementary Fig. 2B). Specifically, Exh T_CD8 did not show significant statistical significance, but the median percentage of Exh T_CD8 tended to be higher in the severe COVID-19 group (Fig. 1F). Notably, the Macro/T cell type was a unique cluster, which was observed in the severe group only (Fig. 1C and F) and expressed the canonical genes of both macrophages (*CD163, MARCO*, and *FCGR1A*) and T cells (*CD2, CD3E*, and *CD3D*) (Fig. 1D). Epi were significantly enriched in both the moderate and severe groups with SARS-CoV-2 infection, and the median percentage in the severe group was higher than that in the moderate group (Fig. 1F).

### Cytokine storm promoting TEX in severe COVID-19 via cytokine-receptor interaction axes

**Figure 2.**
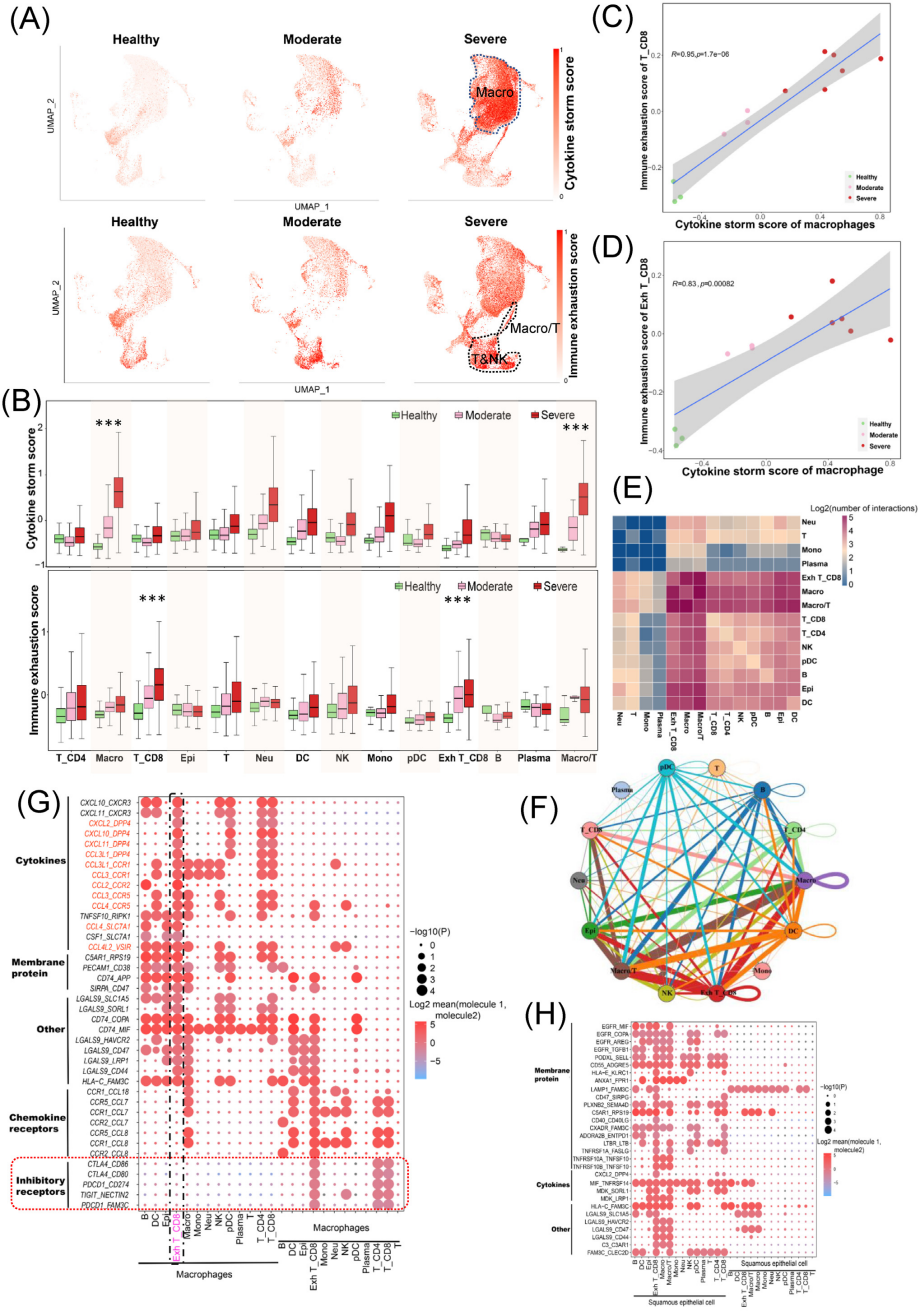
Impacts of cytokine storms derived from macrophages on CD8 T cell exhaustion. (**A**) UMAP plots of BALF cells colored by cytokine storm (top panel) and immune exhaustion (bottom panel) scores across healthy controls (*n* = 3), moderate patients (*n* = 3), and severe patients (*n* = 6). The scores were scaled as 0–1. (**B**) Cytokine storms (top panel) and immune exhaustion (bottom panel) scores. (**C** and **D**) Pearson correlation between immune exhaustion score of CD8 T (T_CD8 and Exh T_CD8 respectively) and cytokine storm score of macrophages. (**E**) Heatmap shows the log2 (transformed number) of interactions between different cell types. (**F**) The cell–cell interaction network shows interaction frequencies between different cell types. Colorful nodes represent cell types, edges stand for cell–cell interaction, and their sizes indicate interaction numbers. (**G** and **H**) Dot plots show the selected ligand–receptor interactions mediating cell–cell communications: (**G**) is for macrophages and other cell types and (**H**) for squamous epithelial cell and other cell types. The circle size indicates *P* value. The means of the average expression levels of interacting molecule 1 in cluster 1 and interacting molecule 2 in cluster 2 are indicated by colour.

Recent studies suggested that cytokine storm was strongly associated with COVID-19 severity, and macrophages may contribute to cytokine storms in BALF.^13^,^26–29^ Therefore, we analysed the single-cell sequencing data of BALF samples infected by SARS-CoV-2, and interrogated the potential relationship between cytokine storm and TEX. We computed the cytokine storm scores based on the expression of eight reported pro-inflammatory cytokines (*IL2, IL7, CSF3, CXCL10, CCL2, CCL3, TNF*, and *IL6*) detected in patients with cytokine storms,^1^ as well as the immune exhaustion scores based on the expression of six inhibitory receptors (*PDCD1, CTLA4, LAG3, BTLA, TIGIT*, and *HAVCR2*). As expected, the macrophages in the severe group had the highest cytokine storm scores among healthy, moderate, and severe groups (Fig. 2A and B), and the cytokine storm scores of macrophages gradually increased across the three groups (Fig. 2B). Surprisingly, the mixture of macrophages and T cells (Macro/T) had both high cytokine storm scores (Fig. 2B top panel) and high immune exhaustion scores (Fig. 2B bottom panel), suggesting that cytokine storm likely promoted the interaction between macrophages and T cells. On the other hand, all Exh T_CD8, T_CD4, and T cells exhibited gradually increasing exhaustion level across healthy, moderate, and severe groups (Fig. 2B bottom panel), indicating that TEX occurred in the moderate group and was more serious in the severe group. Pearson correlation analysis illustrated that with increasing cytokine storm scores of macrophages, TEX level increased as well (Fig. 2C and D). Overall, these results indicated that cytokine storms produced by macrophages promoted TEX in the bronchoalveolar immune microenvironment of severe COVID-19.

Then we explored the potential molecular mechanism via which cytokine storms promoted TEX. Of 719 cells belonging to Macro/T, 152 (21.14%) showed expression of LAG3, which is a typical inhibitory receptor representing TEX (Fig. 1G left). We suspected that this cell type may be a technical artefact known as “doublets” during single-cell RNA sequencing, as it was observed in the severe group only. The percentage of macrophages in the severe group was lower than that in the healthy group, indicating that the doublets may have no association with the high cell mass of macrophages. All of this evidence suggested an underlying interaction of T cells and macrophages, which possibly resulted in the occurrence of the Macro/T “doublets”. Therefore, we performed a cell–cell communication analysis through CellPhoneDB^25^ based on the combined expression of multi-subunit ligand–receptor complexes for the severe group cells. We found that Exh CD8_T cells had higher numbers of interactions with macrophages (Macro) and Macro/T cells (Fig. 2E). Specifically, cytokines (CXCL10, CXCL11, CXCL2, CCL2, CCL3, and so on) expressed in macrophages acted as ligands and significantly interacted with receptors (CXCR3, DPP4, CCR1, CCR2, CCR5, and so on) expressed in exhausted CD8 T cells (Exh T_CD8), forming the cytokine–receptor interaction axes (Fig. 2G). Multiple inhibitory receptors (CTLA4, PDCD1, TIGIT and HAVCR2) expressed in exhausted CD8 T cells significantly interacted with membrane protein genes (*CD86, CD80, CD274/PD-L1*, and *NECTIN2*). Cell–cell interaction analysis indicated that macrophages interacted with Exh T_CD8 cells via the cytokine–receptor axes in the severe group (Fig. 2F). Meanwhile, Epi cells had a high number of interactions with Macro, Macro/T, and Exh T_CD8 cells (Fig. 2F), and many membrane proteins expressed in Epi cells interacted with membrane proteins, cytokines and secretory proteins expressed in Exh T_CD8 cells (Fig. 2H).

### Two macrophage subtypes were the main source of cytokine storms

**Figure 3.**
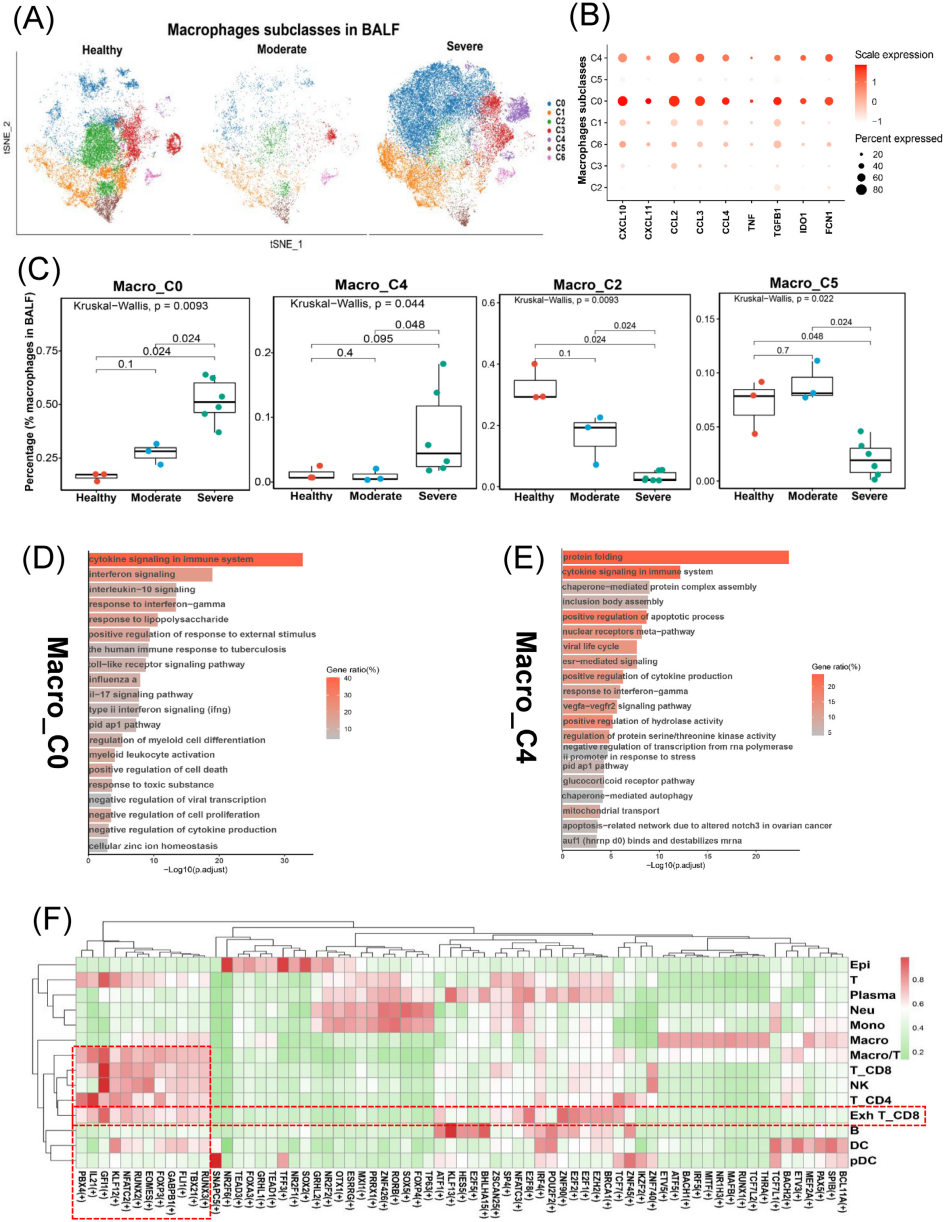
Identification of macrophage subclasses related to cytokine storms. (**A**) tSNE map plot of the macrophages colored by heterogeneous subclasses and split into the three disease states (healthy, moderate, and severe). (**B**) Dot plots show the expression levels of cytokine storms-related genes across 7 macrophage subclasses. (**C**) Boxplots show the cell percentages of 4 macrophage subclasses across three disease states. (**D** and **E**) The top 20 enriched pathways/processes of the top 100 differently expressed genes in Macro_C0 (**D**) and Macro_C4 (**E**) (hypergeometric test, adjusted *P*-values obtained by the Benjamini–Hochberg procedure). (**F**) Heatmap shows the regulon specificity score (RSS) for transcriptional factors (columns) corresponding to cell types (rows).

To further decompose the macrophage heterogeneity and identify macrophage subsets that contributed to cytokine storms, we re-clustered these macrophage cells into six subclasses (Macro_C0 to Macro_C5) (Fig. 3A). Macro_C0 and Macro_C4 were enriched in the severe group (Fig. 3C) and had higher expression levels of cytokine-storm genes (*CXCL10, CXCL11, CCL2, CCL3, CCL4, TNF*, and *TGFB1*) than other subclasses (Fig. 3B). Macro_C2 and Macro_C5 were enriched in the healthy control (Fig. 3C) but lacked expression of cytokine-storm genes (Fig. 3B). This evidence suggested that Macro_C0 and Macro_C4 macrophage subclasses were the main sources of cytokine storms in the bronchoalveolar immune environment. Pathway and process enrichment analysis of the top 100 differently expressed genes (Supplementary Table 4, see online supplementary material) in Macro_C0 and Macro_C4 respectively illustrated that these genes played vital roles in cytokine signalling in the immune system, interferon signalling, and interleukin-10 signalling (Fig. 3D and E).

### Identification of transcription factors related to TEX and cytokine storms

The biogenesis of TEX and cytokine storm relies on the intracellular transcriptional state driven by transcription factors.^30,31^ The transcriptional state of a cell emerges from an underlying gene regulatory network (GRN) in which a limited number of transcription factors (TFs) and cofactors regulate each other and their downstream target genes.^32^ Using pySCENIC, we reconstructed regulons based on the raw count matrix of the severe group and assessed the enrichment score for each regulon's target genes (AUCell). We identified master regulons that were specific to cell types via the regulon specificity score (RSS) (Supplementary Table 5, see online supplementary material). Finally, we predicted that transcription factor genes *TBX21, NFATC2*, and *EOMES*, which were involved in the transcriptional regulatory network for the development of TEX,^33^ were the master regulons with higher RSS for exhausted T cells (Fig. 3F). *GFI1, ZNF90, E2F8, E2F2*, and *EZH2* were also specific to exhausted T cells and may play a vital role in TEX in severe COVID-19. In terms of macrophages, interferon regulatory factor 5 (*IRF5*) and *NR1H3* involved in the inflammation-related transcriptional programs of macrophages^34^ were predicted as master regulons that might regulate cytokine-related gene expression (Fig. 3F).

### Cytokine storms showed no association with TEX in PBMC

**Figure 4.**
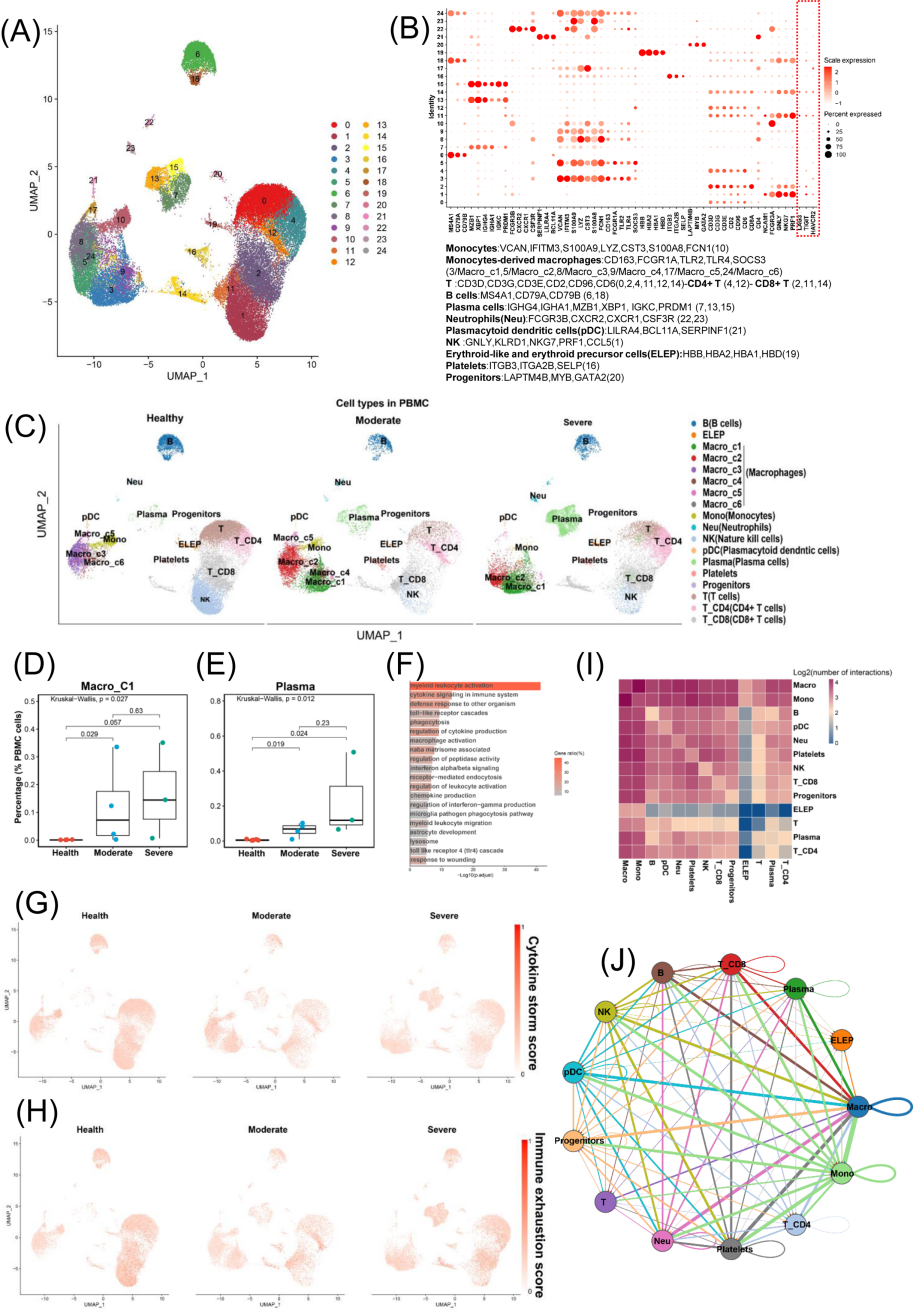
Single-cell RNA-seq analysis to investigate the impact of cytokine storms on TEX in PBMC. (**A**) Overview of the cell clusters in PBMCs (*n* = 13) derived from across healthy people (*n* = 6), moderate (*n* = 4), and severe (*n* = 3) COVID-19 patients. (**B**) Dotplots show the expression level of canonical markers representing cell types in PBMC. Cell types and their corresponding genes are listed at the bottom. (**C**) UMAP plots of PBMC colored by cell type among three disease states (healthy control, moderate, and severe). Cell percentage comparisons of Macro_C1 (**D**) and plasma (**E**) in PBMC across healthy controls, moderate, and severe patients. Kruskal–Wallis H test. (**F**) Pathway and process enrichment analysis of the top 100 highly expressed genes in a subclass of macrophages (Macro_C1). UMAP plots of PBMCs colored by cytokine storm (**G**) and immune exhaustion (**H**) scores across three disease states. The scores are scaled to 0–1. (**I**) Log2-transformed frequency of interactions between cell types of PBMC. (**J**) Cell–cell interaction network shows the interaction frequencies between cell types of PBMC.

To investigate how cytokine storms impact TEX in peripheral blood of patients with COVID-19, we performed scRNA-Seq data analysis of PBMC samples of moderate COVID-19, severe COVID-19, and healthy control. A total of 24 cell clusters were identified, including harboured monocyte, monocytesderived macrophage, T, CD4 T, CD8 T, B, plasma, and NK cells (Fig. 4A and B). We compared the percentage of each cell type and observed that the plasma cells and a subclass of macrophages (Macro_C1) were significantly enriched in PBMC of severe COVID-19 (Fig. 4D and E, and Supplementary Fig. 3B). Pathway and process enrichment analysis of the top 100 highly expressed genes in Macro_C1 showed that these genes were also associated with cytokine-related pathways and processes (Fig. 4F). With respect to TEX, compared with the exhausted T cell cluster which expressed multiple inhibitory receptors in BALF, only a subset of T cells had the expression of *TIGIT, LAG3*, or *HAVCR2* in PBMC (Fig. 4B). We then calculated immune exhaustion scores and cytokine storm scores of PBMC. We found a minority of cells with high immune exhaustion scores or cytokine storm scores, but these cells were not significantly enriched in each of the groups or cell types (Fig. 4G and H). Cell–cell interaction analysis illustrated that macrophages did not have high frequency of interactions with T cells in PBMC of severe COVID-19 (Fig. 4I and J). These results indicated that although a macrophage subclass related to cytokine production occurred in PBMC of severe COVID-19, the macrophages did not cause cytokine storms in PBMC.

### Gene expression of cytokine–receptor interaction axes correlated with poor prognosis in lung squamous cell carcinoma

**Figure 5.**
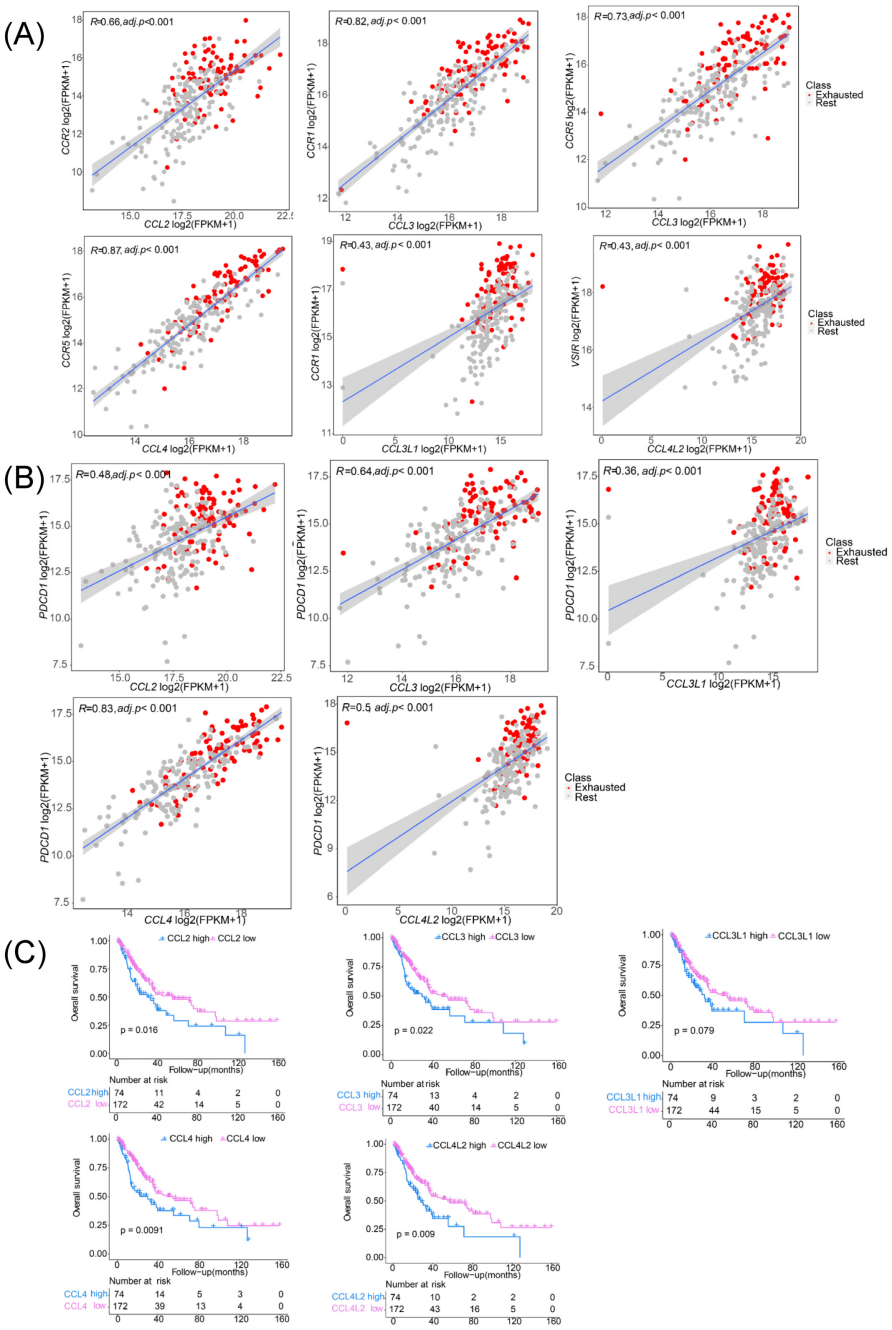
Expression correlation analysis of cytokines and receptors in a LUSC cohort and survival analysis based on cytokine expression level. (**A**) Correlation of expression between the paired genes*CCL4-CCR2, CCL3-CCR1, CCL3-CCR5, CCL4-CCR5, CCL4L2-VSIR*, and *CCL3L1-CCR1*. (**B**) Pearson correlation analyses indicate the strong association of cytokines with PDCD1 expression. (**C**) High expression of CCL2, CCL3, CCL4, CCL3L1, and CCL4L2 is associated with poor overall survival of LUSC patients.

Cytokine storm is a common clinical feature in both severe COVID-19 and lung squamous cell carcinoma (LUSC).^35^ Exhausted T cells are widely present in the tumour microenvironment, leading to immune evasion.^36^ To validate cytokine storm-related axes that impact TEX, we performed expression analysis in a TCGA LUSC cohort to explore the co-expression of cytokines and receptors that formed the interaction axes in severe COVID-19. The paired genes of*CCL2-CCR2* (*R* = 0.66, adjusted *P* < 0.001), *CCL3-CCR1* (*R* = 0.82, adjusted *P* < 0.001), *CCL3-CCR5* (*R* = 0.73, adjusted *P* < 0.001), *CCL4-CCR5* (*R* = 0.87, adjusted *P* < 0.001), *CCL4L2-VSIR* (*R* = 0.43, adjusted *P* < 0.001), and*CCL3L1-CCR1* (*R* = 0.43, adjusted *P* < 0.001) axes were co-expressed in LUSC (Fig. 5A), suggesting that cytokines may also interact with the receptors of T cells and promote TEX in LUSC. Of these cytokine–receptor axes, the CCL2-CCR2 axis has been reported to be involved in immune evasion through PD-1 signalling in esophageal carcinogenesis.^37,38^ In our study, the cytokine genes (*CCL2, CCL3, CCL4, CCL3L1*, and *CCL4L2*) showed correlations with PD-1 (PDCD1) expression (Fig. 5B). Survival analysis based on the grouping of cytokine gene expression levels indicated that the high expression of *CCL2, CCL3, CCL4, CCL3L1*, and *CCL4L2* was significantly associated with poor prognosis in LUSC (Fig. 5C). In brief, these evidences demonstrated that similar cytokine storms promoted TEX in COVID-19 and LUSC, and were associated with poor clinical outcomes. The cytokine-receptor axes may be a potential targets of immunotherapy. The targeted drugs of the cytokine–receptor axes are summarized in Table 2.

**Table 2.** Cytokine–receptor axes identified in this study.

Axis	Cytokine/receptor	Description	Correlation among LUSC cohort	Drug
**CCL3L1_CCR1**	CCL3L1	C-C motif chemokine ligand 3 like 1	*R* = 0.43, *P* = 1.1e−12	No record
	CCR1	C-C motif chemokine receptor 1		DAPTA, BX 471, CCL3 ,CCL4, J 113863
**CCL3_CCR1**	CCL3	C-C motif chemokine ligand 3	*R* = 0.82, *P* < 2.2e−16	ROX-888
	CCR1	C-C motif chemokine receptor 1		DAPTA, BX 471, CCL3, CCL4, J 113863
**CCL2_CCR2**	CCL2	C-C motif chemokine ligand 2	*R* = 0.66, *P* < 2.2e−16	Danazol, Atorvastatin, Simvastatin, Chondroitin sulfate, Risperidone
	CCR2	C-C motif chemokine receptor 2		INCB3284, CCX915, Plozalizumab, MK-0812, DAPTA
**CCL3_CCR5**	CCL3	C-C motif chemokine ligand 3	*R* = 0.73, *P* < 2.2e−16	ROX-888
	CCR5	C-C motif chemokine receptor 5		Maraviroc, Dexamethasone, Efavirenz, Etravirine, Ritonavir
**CCL4_CCR5**	CCL4	C-C motif chemokine ligand 4	*R* = 0.87, *P* < 2.2e−16	No record
	CCR5	C-C motif chemokine receptor 5		Maraviroc, Dexamethasone, Efavirenz, Etravirine, Ritonavir
**CCL4L2_VSIR**	CCL4L2	C-C motif chemokine ligand 4 like 2	*R* = 0.43, *P* = 2.1e−12	No record
	VSIR	V-set immunoregulatory receptor		No record

## Discussion

Cytokine storms involve elevated levels of pro-inflammatory cytokine and immune cell hyperactivation, leading to coagulopathy, multiple organ failure and even death.^1,35^ It has been reported that cytokine storms are associated with TEX.^4,5^ However, knowledge about how cytokine storms impact TEX remains lacking. In this study, we revealed that cytokine storms released by macrophages promoted TEX via CCL2-CCR2, CCL3-CCR1, CCL3-CCR5, CCL4-CCR5, CCL4L2-VSIR, and CCL3L1-CCR1 axes, that are associatied with poor prognosis of COVID-19.

To investigate the interaction mechanism between cytokine storms and TEX, we first analysed single-cell sequencing data of BALF samples of COVID-19 to identify and define 14 cell-type clusters. Of them, the severely exhausted CD8 T cell cluster expressed multiple distinct inhibitory receptors and was enriched in BALF of severe COVID-19. The co-expression of multiple inhibitory receptors was associated with higher exhaustion levels of CD8 T cells and more severe infection during viral infection.^39^ This severe exhausted CD8 T cell type has not been reported by previous studies.^13,26^ Because severe exhausted T cells displayed accumulation of mitochondria,^40^ the low mitochondrial gene percentage parameter setting in the single-cell quality control led to the removal of these cells in previous studies. Consistent with previous studies,^13,26^ Epi cells accounted for a significantly higher cell proportion of BALF in severe COVID-19 compared with that in moderate COVID-19 or healthy controls. Interestingly, the Macro/T cell type which may be “doublets” of macrophages and T cells and expressed exhaustion markers such as LAG3 and HAVCR2, only existed in BALF of severe COVID-19.

We suspected that the interaction of macrophages and T cells caused the presence of Macro/T and was associated with TEX. Therefore, we then conducted cell–cell communication analyses which confirmed the presence of interaction between exhausted T cells and macrophages in severe COVID-19 via cytokine–receptor axes (CXCL2-DPP4, CXCL11-DPP4, CCL3L1-DPP4, CCL3L1-CCR1, CCL3-CCR1, CCL2-CCR2, CCL3-CCR5, CCL4-CCR5, and CCL4L2-VSIR). CXCL10, CXCL11, CCL2, CCL4 and CCL3 were involved in cytokine storms as soluble mediators.^31^ Of these cytokine–receptor axes, CCL2-CCR2 and CCL3-CCR5 have been reported to regulate T cell differentiation.^41^ Our result indicated that cytokine–receptor axes mediate the communication between macrophages and exhausted T cells, impacting TEX.

Further, as reported in previous studies,^42^ cytokine storm was not observed in the moderate group of COVID-19, but was more pronounced in the severe group. While TEX had been observed in moderate patients, subsequently more serious TEX was seen in the severe group. Correlation analysis indicated that cytokine storm level was significantly positively correlated with TEX. This evidence indicated that the presence of cytokine storm promoted TEX in severe COVID-19. Additional cell subtype clustering in macrophages suggested that macrophages might be a key source of cytokine storms in BALF of severe COVID-19.

Blockade of the cytokine–receptor axes mediating the communication between macrophages and exhausted T cells, such as CCL2-CCR2, CCL3-CCR5, and CCL4-CCR5, may attenuate TEX. Clinical trials (NCT04435522, NCT04441385, and NCT04475991) to test the efficacy of maraviroc, a CCR5 antagonist for severe COVID-19, are currently ongoing. Maraviroc may reverse TEX and reduce cytokine storms.^6^ Moreover, dexamethasone, a drug for CCR5 gene (GeneCards GCID: GC03P046383), was reported to be able to reduce 28-day mortality for severe COVID-19.^43^ These findings illustrated that the cytokine–receptor axes may be important therapeutic targets.

Cytokine storms showed no association with TEX in PBMC of COVID-19; however, in lung cancer, especially LUSC, an interleukin-6-related cytokine storm was observed.^35^ In our study, the cytokine storm-related genes and corresponding receptor genes found in severe COVID-19, were significantly correlated with each other in LUSC, and these cytokines had strongly positive correlation with PD-1 expression. This evidence confirmed that a similar cytokine storm was present in LUSC and positively correlated with severe TEX. The co-expression of cytokine and receptor genes (*CCL2-CCR2,CCL3-CCR1, CCL3-CCR5,CCL4-CCR5, CCL4L2-VSIR*, and *CCL3L1-CCR1*) confirmed that cytokine–receptor axes found in COVID-19 may also play a critical role in TEX of LUSC. Of these axes, a previous study demonstrated that the CCL2-CCR2 axis can induce immune evasion via PD-1 signalling and the blockade of this axis can enhance the antitumor efficacy of CD8 T cells in esophageal carcinogenesis.^37^ Anlotinib can induce CCL2 decreases and improve the survival of refractory advanced nonsmall cell lung cancer patients,^44^ suggesting that patients with high CCL2 expression level may benefit from this drug. High expression of cytokine storm-related genes (*CCL2, CCL3, CCL4, CCL3L1*, and *CCL4L2*) was significantly associated with poor prognosis, confirming the prognostic value of cytokine storms in LUSC.

In conclusion, we revealed that cytokine storms may promote TEX through cytokine–receptor axes associated with poor prognosis in COVID-19. Similar findings were verified in LUSC. Blocking the cytokine–receptor axes, such as CCL2-CCR2, CCL3-CCR1, CCL3-CCR5, CCL4-CCR5, CCL3L1-VSIR, and CCL3L1-CCR1, may be a novel strategy to reverse TEX and furhter to treat severe COVID-19 with cytokine storm-related immune exhaustion. Some cytokine–receptor axes (e.g. CCL2-CCR2 and CCL3-CCR2) have been confirmed to be involved in TEX-related immune evasion in other diseases though experiments. However, the molecular relationship between cytokine storms and TEX were not fully investigated experimentally. Through bioinformatics analysis during this study, we provided a new insight into the molecular relationship between cytokine storms and TEX, suggesting a clue to potential cytokine-targeted strategies for immunotherapy and indicating the prognostic significance of cytokine storms.

## Supplementary Material

pbac014_Supplemental_Figures_and_Tables
