# Supplementary material for: Cytokine storm promoting T cell exhaustion in severe COVID-19 revealed by single cell sequencing data analysis
Source: Precis Clin Med. 2022 May 23;5(2):pbac014. doi: 10.1093/pcmedi/pbac014 (PMC9172646; doi:10.1093/pcmedi/pbac014)

**Supplementary Figure 1.** Deep learning-based single cell clustering on the BALF data. (A) The UMAP plot of single cell clusters identified by the scvi-tools based on deep learning model. (B) The UMAP plot of same cell clusters coloured by the cell types from the Seurat analysis.


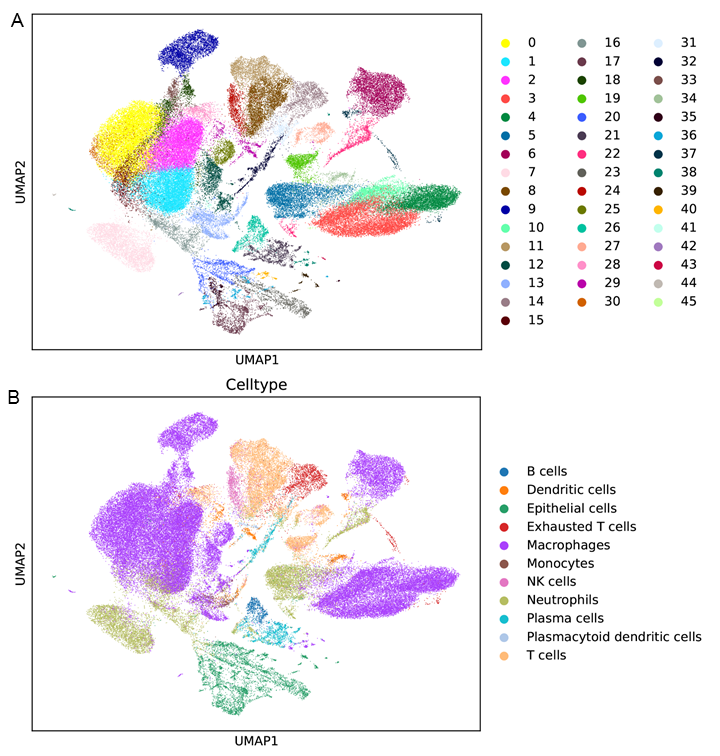


**Supplementary Figure 2.** Single cell RNAseq analysis of BALF related to Figure 1. (A) Elbow plot showing the contribution of each PC to variance. (B) Boxplots showing cell percentages of different cell types among healthy controls, moderate and severe COVID-19 patients


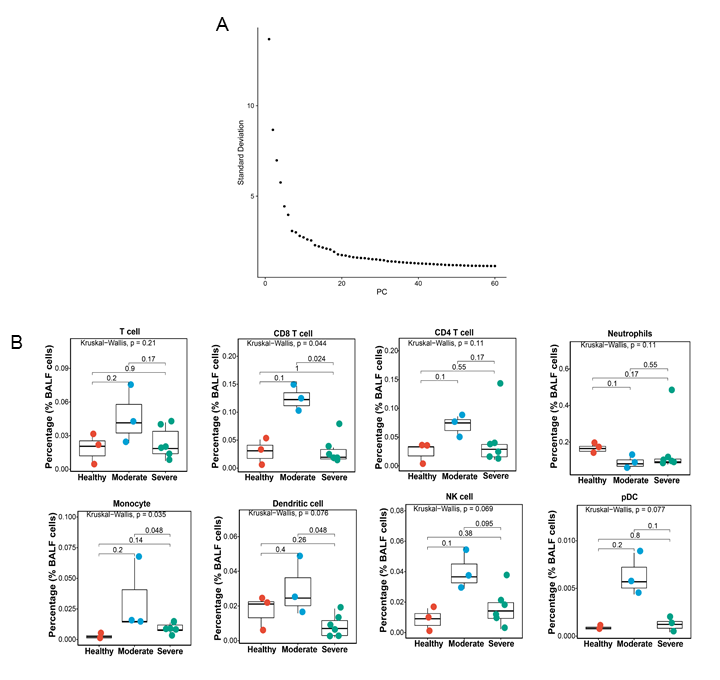


**Supplementary Figure 3.** Single cell RNAseq analysis of PBMC related to Figure 4. (A) Elbow plot showing the contribution of each PC to variance. (B) Boxplots showing cell percentages of different cell types among healthy controls, moderate and severe COVID-19 patients


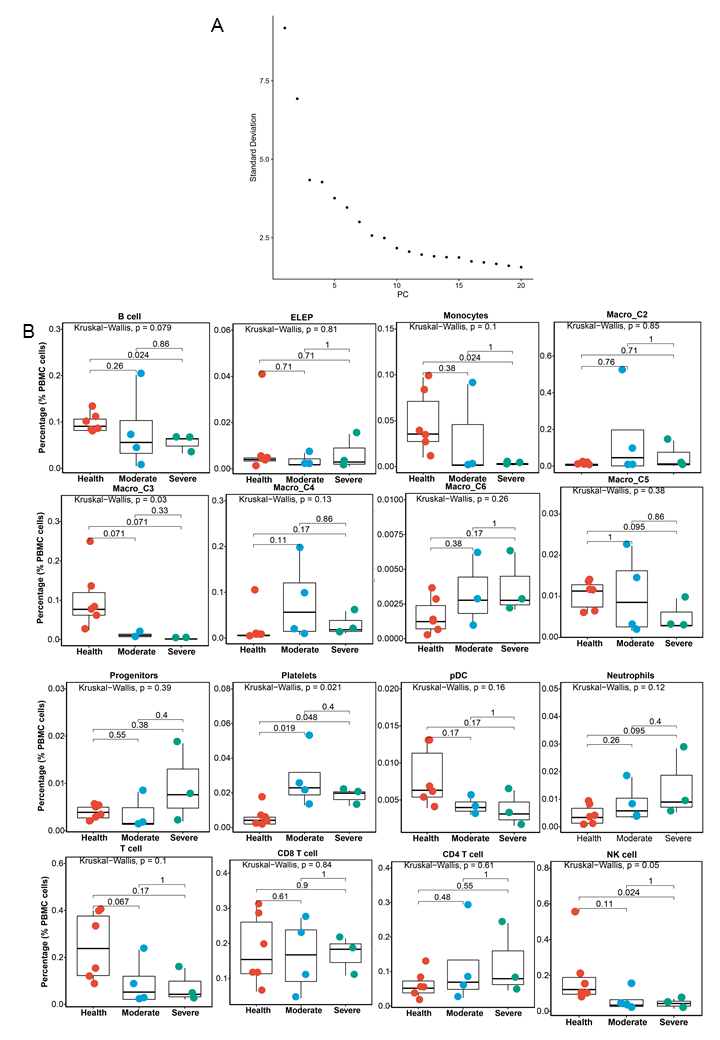

Supplement: pbac014_Supplemental_Figures_and_Tables [file pbac014_supplemental_figures_and_tables.zip › Supplementary Figures.docx]
